# A qualitative systematic review of anonymous/unspecified living kidney and liver donors’ perspectives

**DOI:** 10.1371/journal.pone.0277792

**Published:** 2022-12-30

**Authors:** Wen Hui Lim, Kai En Chan, Cheng Han Ng, Darren Jun Hao Tan, Phoebe Wen Lin Tay, Yip Han Chin, Jie Ning Yong, Jieling Xiao, Clarissa Elysia Fu, Benjamin Nah, Ho Yee Tiong, Nicholas Syn, Kamala Devi, Konstadina Griva, Loey Lung Yi Mak, Daniel Q. Huang, James Fung, Mohammad Shadab Siddiqui, Mark Muthiah, Eunice X. X. Tan

**Affiliations:** 1 Yong Loo Lin School of Medicine, National University of Singapore, Singapore, Singapore; 2 Division of Gastroenterology and Hepatology, Department of Medicine, National University Hospital, Singapore, Singapore; 3 National University Centre for Organ Transplantation, National University Health System, Singapore, Singapore; 4 Department of Urology, University Surgical Cluster, National University Hospital, Singapore, Singapore; 5 Lee Kong Chian School of Medicine, Nanyang Technological University, Singapore, Singapore; 6 Division of Gastroenterology and Hepatology, Department of Medicine, The University of Hong Kong, Pok Fu Lam, Hong Kong; 7 Division of Liver Transplantation, Department of Surgery at Queen Mary Hospital, Pok Fu Lam, Hong Kong; 8 Division of Gastroenterology, Hepatology and Nutrition, Department of Internal Medicine, Virginia Commonwealth University, Richmond, Virginia, United States of America; Imperial College Healthcare NHS Trust, UNITED KINGDOM

## Abstract

**Objectives & background:**

Anonymous live organ donors or unspecified donors are individuals willing to be organ donors for any transplant recipient with whom they have no biological or antecedent emotional relationship. Despite excellent recipient outcomes and the potential to help address organ scarcity, controversy surrounds the unconditional act of gifting one’s organs to an unrelated recipient. This qualitative systematic review provides insights into the first-hand experiences, motivations, and challenges that unspecified donors face.

**Methods:**

A systematic search was conducted on Medline, Embase, CINAHL, PsycINFO, and Web of Science database for qualitative literature regarding unspecified living donors’ motivations and experiences in liver and kidney transplantation. An inductive thematic analysis was conducted to generate themes and supportive subthemes.

**Results:**

12 studies were included. The four major themes were (i) motivations, (ii) perception of risks, (iii) donor support, and (iv) benefits of donation. Unspecified donors demonstrated a deep sense of social responsibility but tended to underestimate health risks in favour of benefits for recipients. Despite the lack of emotional support from family and friends, the decision to donate was a resolute personal decision for donors. Majority benefitted emotionally and did not express regret.

**Conclusion:**

This qualitative review bridges the gap in literature on unspecified living donor psychology and provides a comprehensive understanding of the decision-making matrix and experiences of donors.

## Introduction

Living donors accounted for almost 15% of organ donation in the United States in 2020 [1]. However, growth of the organ transplant waiting list continues to outpace the limited supply of organ donors, and more than 106,000 people remain on the waiting list for lifesaving organ transplantation including but not limited to kidney, liver, pancreas, heart and lung in the United States [2]. Anonymous live organ donors or unspecified donors are individuals who are willing to be organ donors for any potential transplant recipient with whom they have no biological or antecedent emotional relationship [3]. Although the number of transplants carried out using these donors remains relatively small, an upward trend has been observed in recent years which can potentially help mitigate the shortage of liver and kidney grafts globally [4, 5]. In addition, unspecified donations can help bridge transplant disparities [6] and improve the equity of graft allocation by increasing opportunities for waitlist candidates who lack willing donors, including women, racial minorities and those who are financially disadvantaged.

Despite excellent recipient outcomes [7, 8] and the willingness of this unique group of healthy individuals to expose themselves to health risks to offer patients the chance of life, inclusion of unspecified donors in transplantation programs remain controversial with only a limited number of centres that accept non-directed donations [4]. The reluctance to accept unspecified living donors likely results from multiple sources, including unresolved ethical concerns, legal barriers, providers’ scepticism of donors’ motivations, the logistical complexity in developing and maintaining anonymous live organ donor programs, as well as concerns regarding regulatory ramifications and potential for commercialization [9, 10]. The limited literature available report quantitative data on the characteristics and outcomes of unspecified donors and have demonstrated comparable quality of life and psychosocial benefits among this group of living donors [11–14]. Nevertheless, the lack of in-depth understanding of the profile, motivations, and experiences of unspecified donors continues to hinder the expansion of anonymous living organ donor programs across transplant centres. Qualitative studies are better able to capture broader narratives which can uncover the complex interplay of influences on donors’ decision-making process. Rich narrative data may also provide deeper insights into the challenges that donors face to address existing gaps in care. Thus, this study aimed to conduct a thematic synthesis of qualitative studies to shed light on the beliefs and experiences of unspecified living kidney and liver donors to help increase acceptance in the transplant community and inform the development of relevant programs and guidelines.

## Materials and methods

### Search strategy

This qualitative systematic review was conducted in accordance to the Preferred Reporting Items for Systematic Reviews and Meta-Analyses (PRISMA) statement found in S1 and S2 Files and Enhancing Transparency in Reporting the Synthesis of Qualitative Research (ENTREQ) guidelines [15, 16]. Electronic databases including MEDLINE, Embase, CINAHL, PsycINFO, and Web of Science Core Collection were systematically searched from inception till 15 Feb 2022. References of related literature were manually screened to ensure a comprehensive search. The search strategy is attached in S3 File.

### Study selection and eligibility criteria

Two pairs of authors (WHL and CHN; DJHT and YHC) independently screened the titles and abstracts and conducted a full text review. Only original, peer-reviewed studies written in or translated into the English language were considered. The inclusion criteria were as follows: (i) qualitative studies and (ii) those that examined the perspectives and experiences of unspecified living kidney and liver donors. Studies on potential unspecified living kidney and liver donors who did not go through with donation were also included to better reflect actual clinical practice where a substantial portion of donor candidates may fail to be eligible at the end of a long work-up process due to medical or social reasons despite their initial interest to donate. Given that the experiences of unsuccessful donor candidates are often neglected, this comprehensive review sought to take into account the perspectives of these potential donors and synthesize gaps in care specific to this donor population which is critical for the development of a holistic unspecified living donor program. The definition of anonymous live organ donor or unspecified donor is an individual who volunteers to donate their organ to a recipient with whom they had no biologic connection or prior relationship, thereby excluding spouses, friends, colleagues, and family members. As anonymous donation can be ‘directed’ or ‘non-directed’, this study used the term unspecified donor to refer to all anonymous living organ donors [17]. Commentaries, letters to the editor, reviews, conference abstracts, grey literature, case series, and paediatric studies were excluded. Decision on the final inclusion of articles was based on consensus among authors.

### Data extraction and analysis

Thematic synthesis was conducted using the Thomas and Harden framework which involved three stages of detailed data review and synthesis [18]. Primary sources were first extracted by two pairs of independent authors (WHL and CHN; DJHT and YHC) into a structured proforma in a line-by-line fashion to allow interpretation of data in its context. Repeated reading was then conducted to identify preliminary ideas to construct descriptive themes. The concepts were examined for similarities, variations and relationships with one another to inform the development of an analytical schema of higher-order themes through inductive coding. Discussions were held among authors in consultation with senior author (MM) who reviewed the articles independently to ensure that the coding framework and themes captured all the relevant data from the primary studies.

### Quality assessment

Quality appraisal of included studies was conducted by two independent reviewers (DTJH and JNY) using the Critical Appraisal Skills Programme (CASP) [19]. The CASP Checklist consists of 10 items developed to assess the trustworthiness, relevance, and results of published papers. For each study, an assessment of the transparency of reporting was conducted to provide contextual details for the reader to evaluate the credibility, dependability, and transferability of the study findings to their own setting. Both reviewers met regularly to resolve any disagreements in consultation with a third author (MM). The results of the quality assessment did not result in exclusion of any studies but increased the collective rigor of the synthesis.

### Patient and public involvement

There were no patients and public involved in the research except as participants of the study. The study was conducted in accordance with the Declaration of Helsinki. The study was exempt from IRB review as no confidential patient information was involved.

## Results

### Summary of included studies

**Fig 1:**
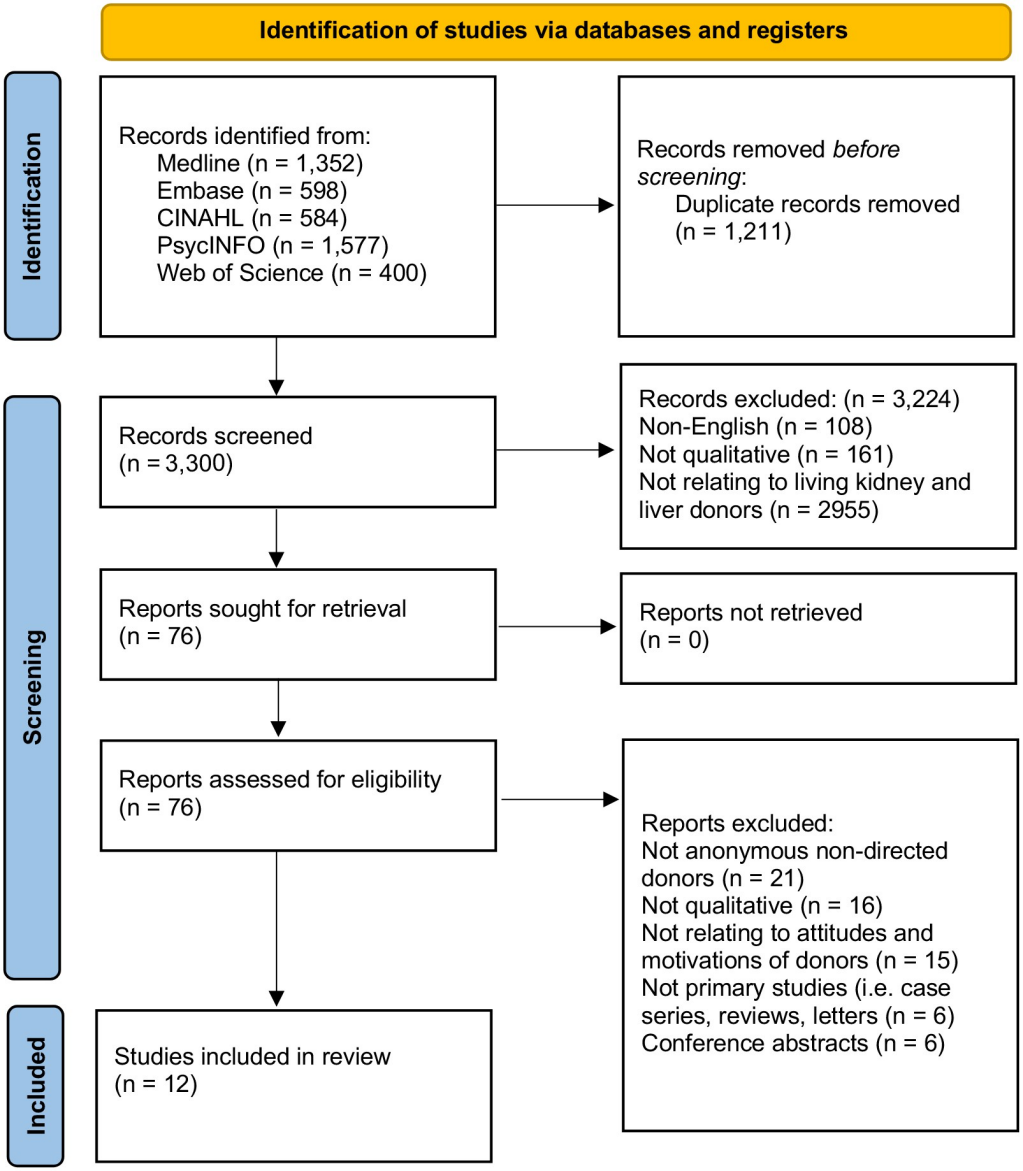
PRISMA flowchart of included articles.

The search strategy yielded a total of 3,300 articles from the electronic databases after the removal of duplicates. A title and abstract sieve was conducted and 76 articles were selected for a full-text review. Of these, a total of 12 articles were included (Fig 1). Studies were conducted in the United Kingdom [20–22], the United States [23–25], Canada [26–28], New Zealand [29], the Netherlands [30], and Sweden [31]. In total, there were 163 unspecified living kidney donors and 26 living liver donors, of whom 129 kidney donors and all 26 liver donors completed donation at time of study. The articles were published between 2010 to 2021 and data were largely collected using semi-structured interviews and focus groups. The study characteristics and quality assessment are summarized in S1 and S2 Tables respectively. The quality of the included studies was largely of no concern (n = 6) or of minor concern (n = 6). Four main themes emerged from the thematic analysis of the included articles. These were (1) motivations, (2) perception of risks, (3) donor support, and (4) benefits of donation. Supporting quotes for individual themes and subthemes can be found in the results section.

### Motivations

#### Decisive moment

“I didn’t even know you could donate a liver. I had no idea any of this was possible. And I read that post [on Facebook] and I thought ‘Oh my God, ok, I’m the same blood type, so you need to help” [26].

Many participants were prompted to donate by a specific event or person. These reasons included emotive media stories about transplant recipients, knowing of a transplant recipient who felt great, hearing about examples of successful transplants, coping with a death in the family, or surviving a personal accident [20, 21, 23, 26, 29–31]. Participants described an instantaneous and emotional response to learning about the possibility of living, non-related donation [20, 21, 26, 29].

#### Alignment with donor identity

"I wanted to contribute to solving the shortage of organ donors. I have been a blood and bone marrow donor for 8 years. Donating a kidney went a step further but fits with my mind-set about being a donor” [30].

The act of organ donation was perceived by many as a natural extension of their identity. Participants drew parallels with being blood or bone marrow donors and shared about other past altruistic acts, such as volunteering or their church mission work [20, 21, 23, 28, 30, 31]. Some participants also cited religion as a motivating factor [23, 25, 29–31]. Others however viewed organ donation as a means of leaving behind a positive legacy to reconcile their sense of self [21, 26, 28].

#### Deep sense of social responsibility

“It might not have a huge impact on my life but could have such a life-changing impact on one person or even if you have initiated a chain of donations, then several people’s lives? In terms of risk versus reward, the way I saw it was I was very much interested in doing it and wanted to find out more” [22].

Participants reported a strong will to help and do good and unspecified donation was construed as a way of significantly impacting the lives of others [20, 21, 23, 26, 29–31]. Donors often wanted to give back to society in appreciation for their fortunate circumstances and expressed that non-directed organ donation provided the opportunity to offer an unconditional gift without expectations of reciprocity [21, 26, 29]. Some also mentioned the prospect of a donation chain which could exponentially help more patients [22, 23].

### Perception of risks

#### Fears and concerns

“Oh one it kept going back to… because I have a little sister who is very young, ‘what if your little sister needs it and you have already given it away to some stranger?’ and all that sort of thing. Or even my dad, who is over 60 now, was like ‘what if I need it?’ And obviously I get that like, family is obviously very important but I just always thought that giving your kidney to anyone is a fantastic thing to do, whether you are related to them or not, so I really thought it was kind of saving a life,… the chances of one of them also needing a kidney donation was so slim, but if it did happen then maybe they would be able to be donated a kidney by somebody else doing the same thing I did” [22].

Participants acknowledged fears surrounding organ donation which included potential sacrifices to lifestyle, surgical risks, long term health effects, the possibility of graft failure in the recipient, and the risks that a family member would need an organ in the future or the participant themselves might acquire a medical condition that would result in a need for an organ transplant [20, 22, 23, 25]. However, most participants weighted their decision in favour of the positive impact on the recipient [21, 22, 29, 30].

#### Fatalism

“If I gave a kidney away tomorrow and in 12 months’ time I had problems with the one that I had left which ended up resulting in me dying, I know this sounds probably a little bit depressing but I did what I could with the information that I had at the time” [21].

Some participants expressed their willingness to accept the risk of mortality, opposing the view that fear of death should impede their decision-making as risks exist in daily life and felt that one should not worry about events beyond their control [21, 22, 29].

#### Trust in the medical system

“I really do trust the doctors, they’re not going to do something that’s wrong” [21].

Participants felt confident in the medical system with some expressing minimal fear regarding the surgical risks. They trusted in the competency of the medical team and believed that the benefits of a successful transplant in affording recipients a chance at life outweighed the risks to themselves [21, 23, 29]. Some participants expressed personal preferences in terms of recipient selection such as donating to a recipient who would take care of their body and lead a healthy lifestyle, but most valued graft compatibility and improving recipients’ quality of life, leaving the medical team to select the most suitable recipient [23, 26, 29].

#### Confidence in physical resilience

“I just imagine the body as like a vehicle, right? Some people are dealt a lemon and if I have a spare part that can be helpful for someone else’s lemon, then I’m going to share it. That’s how I was thinking of it, this is my vehicle and we have this technology for a reason, so why not?” [26].

Majority of participants were confident of their own physical resilience and good health and hence felt they could overcome the risks associated with donation [22, 29]. The donated organ was often viewed as a spare part and many participants believed that they would not be more susceptible to health problems and could live well after donation [22, 26, 29, 31].

### Donor support

#### Lack of emotional support

“… the most surprising thing about the whole thing is the reaction of people when I tell them. I started telling friends and I would say 80% of friends say that I’m mad and some of them are quite vocal about it and think it’s completely the wrong thing to do and you’re messing with nature, and if, you wouldn’t have two kidneys if you didn’t need two kidneys, and what if your children need them …” [21].

The dominant reaction of family and friends was disapproval or active opposition and donors were largely perceived as being either ‘mad’ or ‘brave’ which resulted in feelings of frustration and embarrassment [20–23, 26, 27]. Donors reported that their families were worried and feared for the donor’s life and safety [20, 27]. Conversely, some stated that their families were supportive and emphasized the importance of emotional support from their loved ones during the donation process [22, 26–28]. Participants were also worried that their motives for donation would be misinterpreted by others as pathological or self-serving [20, 26]. In addition, some donors reported dissuasion from the transplant team which was confusing and distressing [20, 22]. Nevertheless, most participants were generally not deterred by negative response, and many insisted that donation was a resolute personal decision [20, 22, 23, 29].

#### Work-up process

"It’s a lot more stressful during the process of preparation than almost the operation itself" [32].

Participants felt that the research and preparation process equipped them with comprehensive information which strengthened their confidence in their decision to donate [20, 23]. However, some commented that the intensive work-up process was long, stressful, and challenging [20, 23, 29]. The psychiatric assessment was also described as the source of most concern and participants reported feelings of trepidation and vulnerability [20, 29]. Several participants also commented on employer support and financial support as important facilitators of organ donation [24, 26, 29], with suggestions of reimbursement of lost income to alleviate financial burdens [24].

#### Gaps in care

“It wasn’t ‘you’re not going to be able to donate yet, just you’re not ever going to be able to donate because we found something that is going to stop you.’… what they found was that in the platelet cells there was what they call a genetic mutation… you are no longer to do with what you were to do with and they have not washed their hands of you but they have moved on, but it’s all been a bit traumatic to put it mildly” [22].

Participants who found out they were ineligible to donate during the work-up process expressed immense disappointment with some giving feedback that the lack of a proper closure made them feel as if they were disregarded by the transplant team [22, 23, 26]. In addition, while most participants reported a fast post-transplant recovery and were able to return to their original lifestyles fairly quickly [22, 23, 29], there were also patients who had to contend with post-surgery complications [28, 29] and felt that nursing care needed to be more attentive to their needs [29].

### Benefits of donation

#### Empowerment and satisfaction

‘‘It’s a hugely, powerful motivational thing to know that you have changed somebody’s life. It’s powerful, it is like a drug” [29].

There was a positive impact on donors’ sense of self-worth and many reported feelings of fulfilment, contentment, and pride [20–22, 26, 28–30]. Some stated that overcoming the challenges of donation shattered their existing understanding of their personal limitations and gave them courage to imagine new possibilities for themselves [20, 26, 28]. Others gained deep satisfaction from the idea of leaving behind a positive legacy [21, 28]. Majority did not express regret [26, 29, 30] and some donors became advocates for organ donation to inspire others to do the same [22, 23, 30].

#### Connectedness

‘‘I wonder how they are. I wonder if they’re getting that second lease on life and if they’re taking advantage of it and if they’re pushing their own boundaries or, you know, what they’re experiencing” [26].

Some were curious about the surgical outcomes and health of their recipient [26, 29]. However, regardless of outcomes, participants stated that the donation experience allowed them to forge closer emotional connections with recipients, family members, and with the community at large [20, 26–29]. Participants who received appreciation from their recipient felt a deep emotional resonance [20, 29] while donors who did not hear back from recipients expressed disappointment and felt a weaker sense of emotional attachment [20–22, 30].

## Discussion

**Fig 2:**
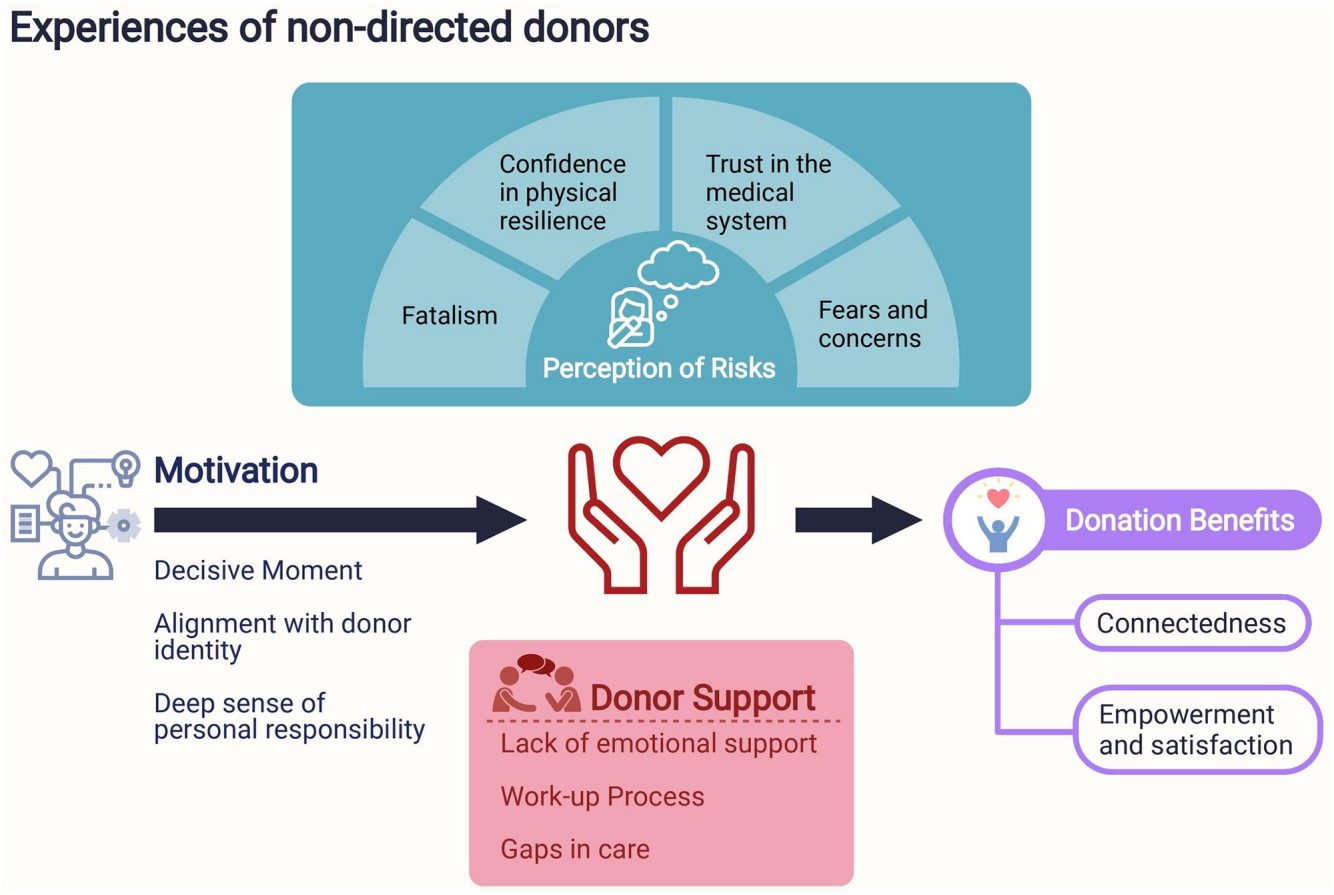
Thematic scheme.

Globally, there is a severe shortage of deceased donor organs, resulting in high mortality rates on liver and kidney transplant waiting lists ranging from 5–25% in Europe and North America [33–35]. Unspecified donations have become an important source to increase the donor pool and is a growing trend, making up 5% and 9% of living kidney and liver donations in the United States in 2018 and 2019 respectively [4, 5]. However, many transplant centres still do not accept unspecified living donors, and controversy continues to surround the unconditional act of gifting one’s organs to an unrelated recipient [9]. The present analysis thus provides insights into the first-hand experiences, motivations, and challenges that unspecified donors face (Fig 2) by synthesising primary qualitative evidence. This will serve to inform the current debate on unspecified organ donation and facilitate the development of future donor programs and refinement of those already in place.

Most unspecified donors believed that donation would result in minimal harm to their health and were willing to accommodate these risks in favour of benefits for the recipient. These beliefs and actions were motivated by donors’ personal experiences with acts of benevolence, their core value of altruism, and a deep sense of social responsibility and compassion. This was consistent across both living kidney and liver donors. A previous qualitative review similarly demonstrated that living donors were driven by a sense of duty irrespective of the organ donated and medical discrepancies regarding procedures and risks between kidney and liver donation did not affect decision to donate [36]. However, there remains a paucity of data regarding long-term donor outcomes, and current studies have largely been limited by short follow-up durations, high loss to follow-up, and inadequate sample size [26, 37]. A recent study by Choi et al. found that living liver donors had twice the all-cause mortality of matched healthy controls despite there being few deaths [38]. Living kidney donation has also been reported to increase the risks of end-stage renal disease, decreased kidney function, hypertension, and proteinuria [39]. Long-term prospective studies on living donor outcomes are therefore urgently warranted to enable more informed decision-making [40]. Of note, ongoing efforts are being undertaken to address this gap in literature by teams in the United Kingdom [41].

The discourse of participants revealed a lack of awareness regarding the potential adverse effects of organ donation on psychological wellbeing. While our findings showed that many participants gained positive emotional benefits from the donation experience, previous studies have reported devastating effects of recipients’ death on donors’ psychological wellbeing, albeit the lack of biological relationship. A substantial increase in depression risk of 123% was reported among unrelated (non-spousal and non-biologically related) donors whose recipients died [42]. Most unspecified donors under-anticipated potential health burdens, highlighting the need for better information transmission and education on the uncertainty surrounding donor safety. These findings further emphasize the importance of careful psychosocial evaluation to assess resilience, mental stability and stress threshold of donors [43–45] and the need to adequately prepare donors for the possibility for graft failure. Currently, the Organ Procurement and Transplantation Network (OPTN) policy requires transplant centres in the United States to follow up with living donors for 2 years with mandated reporting every 6 months(3). Besides proposals of wellbeing programs for living donors [46, 47], further recommendations have been made for unspecified donors to be followed by the transplant community in a formalized registry. One such model is the Living Donor Collective Pilot Registry which currently enrols 6 liver and 10 kidney transplantation programs in the United States [48].

One of the major barriers that unspecified donors faced was opposition and negative judgement from family and friends. While previous studies have emphasized the importance of social relationships in unspecified donation [20], the lack of perceived social support may not be a contraindication as most donors were motivated by genuine altruism and possessed a strong sense of autonomy and determination. Instead, the transplant team should encourage donors to discuss intention to donate with family early on to flag out potential concerns that may prove decisive. In addition, the medical team should consider linking up potential donors with those who have successfully completed donation to provide additional emotional support. Likewise, participants reported negative attitudes among some medical professionals towards unspecified donors. This may arise from reservations held by healthcare workers in view of subjecting fit and healthy people to health risks through live organ donation, which differentiates unspecified donors from other surgical patients. Moreover, unspecified donors may also encounter negative attitudes from healthcare workers more frequently than biologically-related donors as support from the public and various groups of health-care workers for living-unrelated donors has been shown to be significantly lower than living-related donors [49–51]. This points to the need for standardized training programs for transplant staff to improve communication skills and address subconscious prejudices [52]. Better communication with donors is required to justify the rigorous work-up process that is necessary to prevent harm to unspecified donors, although this may sometimes be perceived as onerous.

An important implication of unspecified organ donation is the potential to initiate ‘donor chains’ where incompatible donor-recipient pairs can be circumvented, maximizing the capabilities and efficiency of living donor programs [53–55]. This was raised by some participants as a meaningful way to extend their impact and indicates the importance of eliciting donors’ perspectives and willingness to participate. Given that media appeals and awareness campaigns were shown to play a key role in participants’ decision-making, additional publicity and upfront education of all donor options including ‘donor chains’ may reach out to more potential donors [56] while minimizing future pressures in the event of incompatible matches. Interest in this field has driven the development of a formalized liver paired exchange pilot program in the United States under the United Network for Organ Sharing [57, 58].

Our study also revealed gaps in care for potential donors who did not complete donation process due to ineligibility to donate. For instance, potential donors were mentally unprepared to face the possibility that the work-up process may unveil a previously unknown medical condition as donors largely perceived themselves to be in good health. Similarly, healthcare providers were not well-equipped to follow-up with potential donors who received new medical diagnoses and failed to provide quality care for unsuccessful donor candidates. This often resulted in negative feelings such as disappointment or anger when candidates were unable to go through with the donation. Better communication from the transplant team is needed to adequately prepare potential donors for such an outcome and to ensure that potential donors understand and willingly undertake the risks of uncovering health issues during the work-up process. Follow-up protocols should also be established to ensure that potential donors receive appropriate medical care and gain a sense of closure. Importantly, our findings emphasize the importance of extending empathy and acknowledging the experiences of this group of potential unspecified donors who are often neglected.

### Strengths and limitations

This qualitative review bridges a gap in literature on unspecified living donor psychology and provides a comprehensive understanding of the decision-making matrix and experiences of this unique group of patients. However, limitations should be taken into consideration when interpreting these results. There was a paucity of literature from Asia with most studies originating from North America and Europe even though living donor transplants remain the prevalent source of donor organs in Asia due to the lack of deceased organ donations. Given that unspecified donors from Asia may face a different set of cultural and social challenges, more studies should be conducted to understand the experiences of these donors. In addition, comparisons to elucidate the differences in experiences of liver donors versus kidney donors were limited owing to the sparsity of data on unspecified liver donors. Notwithstanding, thematic analysis revealed consistent findings across both unspecified liver and kidney donors who reported similar motivations to donate irrespective of organ donated similar to a previous review by Kisch et al. [36].

## Conclusion

Living donors will become increasingly relevant in the global transplant community as we head into the next decade. This study showed that many unspecified donors possessed genuine compassion, benefitted emotionally from the experience, and donated without regret. Nevertheless, more safeguards and policies need to be put in place to ensure donor safety and facilitate informed decision-making. Through global collaborations and continued effort to craft donor-centric programs, unspecified living donations may represent the next frontier in helping bridge the gap between organ scarcity and expanding waiting lists.

## Supporting information

S1 File PRISMA abstract checklist. (DOCX)

S2 File PRISMA checklist. (DOCX)

S3 File Search strategy for MEDLINE. (DOCX)

S1 Table Summary of key characteristics in included articles. SOT; Solid Organ Transplantation.
(DOCX)

S2 Table Consolidated Criteria for Reporting Qualitative Health Research (COREQ) for included articles. (DOCX)
