# Supplementary material for: A qualitative systematic review of anonymous/unspecified living kidney and liver donors’ perspectives
Source: PLoS One. 2022 Dec 30;17(12):e0277792. doi: 10.1371/journal.pone.0277792 (PMC9803135; doi:10.1371/journal.pone.0277792)
Supplement: S3 File — (DOCX) [file pone.0277792.s003.docx]

**S3 File. Search strategy for MEDLINE**

1. Organ Transplantation/ or Kidney Transplantation/ or Liver Transplantation/ or ((liver* or hepat* or solid organ or renal or kidney*) adj3 (transplant* or graft*)).tw.
2. Living Donors/
3. 1 AND 2
4. ((non-directed or non directed or altruis* or indirect* or unrelated or unspecifi* or anonym* or unconditional or nonbiological or stranger or community or good samaritan) adj3 (donat* or donor*)).tw.
5. 3 OR 4
6. exp Attitude to Health/ or communication/ or decision making/ or Health Knowledge, Attitudes, Practice/ or (donor* adj2 (perspective* or reflection* or view* or experience* or motivation* or value* or belie* or barrier* or challenge* or difficult*)).tw.
7. exp Qualitative Research/ or ((("semi-structured" or semistructured or unstructured or informal or "in-depth" or indepth or "face-to-face" or structured or guide) adj3 (interview* or discussion* or questionnaire*)) or (focus group* or qualitative or ethnograph* or fieldwork or "field work" or "key informant")).ti,ab. or interviews as topic/ or focus groups/ or narration/
8. 6 or 7
9. 5 and 8
10. Limit 9 to English
