# Supplementary material for: A qualitative systematic review of anonymous/unspecified living kidney and liver donors’ perspectives
Source: PLoS One. 2022 Dec 30;17(12):e0277792. doi: 10.1371/journal.pone.0277792 (PMC9803135; doi:10.1371/journal.pone.0277792)
Supplement: S1 Table — SOT; Solid Organ Transplantation. (DOCX) [file pone.0277792.s004.docx]

**S1 Table. Summary of key characteristics in included articles**

| **Author** | **Year** | **Country** | **SOT type** | **Sample size** | **Response rate (%)** | **Completed donation** | **Data collection** | **Analysis** | **Study topic** |
| --- | --- | --- | --- | --- | --- | --- | --- | --- | --- |
| Humar, S. S., et al. | 2021 | Canada | Liver | 26 | 63.42 | 26 | Semi-structured interview | Content analysis | Role of family in donation experience of non-directed donors |
| Zuchowski et al | 2021 | United Kingdom | Kidney | 35 | 94.59 | 15 | Semi-structured interview | Thematic analysis | Experiences of non-directed donors |
| Krause, S., et al. | 2020 | Canada | Liver | 26 | 65.00 | 26 | Semi-structured interview | Grounded theory | Post-traumatic growth in non-directed donors |
| Maghen, A., et al. | 2018 | United States | Kidney | 31 | 40.79 | 31 | Semi-structured interview | Grounded theory | Spirituality and religiosity of non-directed donors |
| Maghen, A., et al. | 2021 | United States | Kidney | 31 | 40.79 | 31 | Semi-structured interview | Grounded theory | Financial concerns of non-directed donors |
| Wadstom et al | 2019 | Sweden | Kidney | 24 | 92.31 | 24 | Interview & Survey | - | Motivations of non-directed donors |
| Goldaracena er al | 2019 | Canada | Liver | 26 | 63.42 | 26 | Semi-structured interview & Survey | Grounded theory | Motivations and experiences of non-directed donors |
| Balliet et al | 2019 | United States | Kidney | 11 | 100.00 | 2 | Focus groups | Grounded theory | Motivations of non-directed donors |
| Challenor et al | 2014 | United Kingdom | Kidney | 6 | 87.50 | 0 | Interview | 6-stage Foucauldian-inspired analytic strategy; Discourse analysis | How non-directed donors construct their altruism |
| Clarke et al | 2014 | United Kingdom | Kidney | 14 | 93.33 | 14 | Interview | Grounded theory | Donation experience of non-directed donors |
| Tong et al | 2012 | New Zealand | Kidney | 18 | 100.00 | 19 | Semi-structured interview | Grounded theory; Thematic analysis | Self-reflections of non-directed donors |
| Massey et al | 2010 | Netherlands | Kidney | 24 | 96.00 | 24 | Semi-structured interview & Survey | Thematic analysis | Motivations of non-directed donors |

Legend: SOT; Solid Organ Transplantation
