# Supplementary material for: A qualitative systematic review of anonymous/unspecified living kidney and liver donors’ perspectives
Source: PLoS One. 2022 Dec 30;17(12):e0277792. doi: 10.1371/journal.pone.0277792 (PMC9803135; doi:10.1371/journal.pone.0277792)
Supplement: S2 Table — (DOCX) [file pone.0277792.s005.docx]

**S2 Table. Consolidated Criteria for Reporting Qualitative Health Research (COREQ) for included articles**

| Study | Balliet et al (2019) | Clarke et al (2014) | Challenor et al (2014) | Tong et al (2012) | Zuchowski et al (2021) | Goldaracena et al (2019) | Massey et al (2010) | Wadstom et al (2019) | Maghen, A., et al (2021) | Humar, S. S., et al (2021) | Krause, S., et al (2020) | Maghen, A., et al (2018) |
| --- | --- | --- | --- | --- | --- | --- | --- | --- | --- | --- | --- | --- |
| **Personal characteristics** |  |  |  |  |  |  |  |  |  |  |  |  |
| Researcher identified | • | • | • | • | • | • | • | • | • | • | • | • |
| Credentials | • | • | • | • | • | • | • | • | • | • | • | • |
| Occupation | • | • |  | • |  |  | • |  |  | • | • |  |
| Gender | • | • | • | • | • | • | • | • | • | • | • | • |
| Experience and training |  | • | • |  | • |  |  | • | • | • | • | • |
| Relationship with participants | • |  | • |  | • |  |  | • |  |  |  |  |
| **Participant’s selection** |  |  |  |  |  |  |  |  |  |  |  |  |
| Sampling | • | • | • | • | • | • | • | • | • | • | • | • |
| Method of approach | • | • | • | • | • | • | • | • | • | • | • | • |
| Sample size | • | • | • | • | • | • | • | • | • | • | • | • |
| No. and reason for non-participation | • | • |  | • | • | • | • | • | • | • |  | • |
| **Setting** |  |  |  |  |  |  |  |  |  |  |  |  |
| Setting of data collection | • | • | • | • | • | • | • | • | • | • | • | • |
| Presence of non-participants |  | • |  |  |  |  |  |  |  |  |  |  |
| Description of sample | • | • | • | • | • | • | • | • | • | • | • | • |
| **Data collection** |  |  |  |  |  |  |  |  |  |  |  |  |
| Interview guide | • | • | • | • | • |  | • | • | • | • | • | • |
| Repeat interviews |  |  |  |  |  | • | • |  | • | • |  | • |
| Audio/visual recording | • | • | • | • | • |  |  | • | • | • | • | • |
| Field notes | • | • | • | • | • |  |  |  | • | • | • | • |
| Duration | • | • | • | • | • | • |  |  | • | • | • | • |
| Data or theoretical saturation | • | • | • | • | • | • |  |  | • | • | • | • |
| Transcripts returned to participants | • | • | • |  |  |  |  |  | • |  | • | • |
| **Data analysis** |  |  |  |  |  |  |  |  |  |  |  |  |
| No. of data coders | • | • | • | • | • | • | • | • | • |  | • | • |
| Description of coding tree | • | • | • | • |  | • | • |  | • |  | • | • |
| Protocol for data preparation and transcription | • |  | • | • | • | • | • |  | • |  | • | • |
| Use of software | • |  |  | • | • |  |  |  | • |  | • | • |
| Reporting | • | • | • | • | • |  | • | • | • | • | • | • |
| Respondent quotations provided | • | • | • | • | • | • | • | • | • | • | • | • |
| Range and depth of insight into attitudes to organ donation | • | • | • | • | • | • | • | • | • | • | • | • |
| Range and depth of insight into cultural diversity | • | • | • | • | • | • | • | • | • | • | • | • |
| **Total Score** | 25 | 24 | 23 | 23 | 23 | 18 | 19 | 18 | 25 | 21 | 24 | 25 |
